# Prevalence and factors associated with modern contraceptive discontinuation among reproductive age group women, a community based cross-sectional study in Humera town, northern Ethiopia

**DOI:** 10.1186/s12905-018-0663-4

**Published:** 2018-11-22

**Authors:** Nigisti Belete, Ayalnesh Zemene, Hadgay Hagos, Abere Yekoye

**Affiliations:** 1 Department of Midwifery, Axum University College of Health Science, Axum, Ethiopia; 2 St. Paul Millennium Medical College, Addis Ababa, Ethiopia; 3 Department of Midwifery, Mekelle University College of health Sciences, Mekelle, Ethiopia

**Keywords:** Humera, Modern contraceptive, Discontinuation, Reproductive age women

## Abstract

**Background:**

Contraceptive prevalence rate (CPR) for married women aged 15–49 in Ethiopia is 36%, with 35% using modern methods and 1% using traditional methods. However, the discontinuation rate is fairly high. Women usually discontinue contraception use for fertility and method related reasons without adopting an alternate method which in turn leads to many health risks such as unwanted pregnancy, unplanned childbearing, miscarriage, abortion, leads to morbidity and mortality among mothers and newborns. The purpose of this study was to determine the prevalence of modern contraceptive discontinuation and to identify predicting factors.

**Methods:**

A community- based cross- sectional study was conducted in Humera town among 321 married women of reproductive age (15–49 yrs.) who had a history of modern contraceptive use. Systematic sampling technique was employed to select study participants and data was collected by BSc health extension workers using interviewer -administered questionnaire. EPI-INFO (V-7) and SPSS (V-23) software were used for entry and analysis respectively. Descriptive statistics and logistic regression analysis were used to present results accordingly. P- Value < 0.05 was used as a cut point for statistical significance.

**Results:**

The magnitude of modern contraceptive discontinuation was 27.1%. Number of desired children (AOR = 2.83 95% CI = 1.16, 6.89), experience of side effects (AOR = 3 95% CI = 1.2, 7.58), discussion with female friend (AOR = 3.26 95% CI = 1.27, 8.36), counseled on side effects (AOR = 6.55 95% CI = 2.21, 19.39), number of male children (AOR = 2.51 95% CI = 1.06, 5.96), absence of husband support (AOR = 12.99 95% CI = 4.59, 36.78) and presence of community prohibition (AOR = 6.88 95% CI = 3.05, 15.51) were identified as predicting factors for modern contraceptive discontinuation.

**Conclusion:**

Magnitude of modern contraceptive discontinuation among reproductive age group women in Humera was relatively high. Increasing community awareness, involving partners and pre dispensation counseling might help to reduce discontinuation and its consequences. Various targeted messages are also needed to dispel misconception at community level.

## Background

Contraceptive discontinuation, measured as the percentage of currently married women who used a method of contraception in the past but were not using a method at the time of data collection. The discontinuation rates are highest in sub-Saharan Africa where the majority of women in 13 of 18 countries have discontinued using contraception. In countries outside of sub- Saharan Africa, the discontinuation rates vary between 19 and 36% [1].

Demographic and health survey (DHS) reports of different countries on contraceptive discontinuation, reasons, challenges, and solutions among married women 15–49 shows 12-month discontinuation rates were in Kenya 36%, Zimbabwe 17.7%, Armenia 30.6%, Egypt 32% and Colombia 43.8% [2]. Literatures report different reasons (method and fertility related) and factors to discontinue specific methods, where the major factors reported include: Age of participants, parity, family size, having television and radios, decision maker to use modern contraceptive (MC), partner supports, perceived benefit to the family, perceived MC harm, duration of contraceptive use, counseling, desired number of children and type of contraceptive were associated with contraception discontinuation [3–7].

In Ethiopia, use of any contraceptive methods among married women has increased nearly six- fold in the last 20 years, the increase in contraceptive use is especially pronounced for the use of modern methods. Overall, 36% of married women are using a method of family planning; 35% are using MC and 1% are using a traditional method. Despite all this, the discontinuation rate for all methods is 37% [5, 6].

Moreover; women discontinue using modern methods without adopting alternate method for different reasons and this usually leads to many undesirable health risks such as unwanted pregnancy, unwanted or unplanned childbearing, miscarriage, abortion, morbidity and mortality among mothers, newborns or both [7], but knowing the magnitude and predicting factors will help to reduce the burden of these consequences. In addition, very little is known about the prevalence and associated factors modern contraceptive discontinuation (MCD) in Ethiopia in general and no data in the study area in particular. Therefore, this paper aims to have certain contribution in closing this gap.

## Methods

### Study area and study period

This study was conducted from 1 to 30 Feb 2017 G.C in Humera Town; Western Tigray, Ethiopia. Humera is located at 991 km, North-West from Addis Ababa and 580 km western from Mekelle. According to the 2007 Census, the total population of the town is about 34,295 and approximately half of the inhabitants are younger than 20 years old. The town has one general hospital (Kahsay Abera Hospital), one health center (Setit primary health center) and 6 private clinics to serve this population. The health service coverage of the town is 100% and 62% contraceptive prevalence rate.

### Study design and sampling

The study design was community-based cross-sectional study. The sample size was calculated using single population proportion formula with the following assumptions: The proportion of women who discontinued contraception in Agarfa District (25.5%) (23), 95% confidence level and 5% margin of error. The minimum desired sample with this assumption was 292**.** By adding 10% non-response rate, a total of 321 study participants were included in the study.

### Sampling technique

Systematic sampling was used to select study participants. All four kebeles were included and the total number households (HH) of source population in each kebele were identified by number in advance. Then, the final sample size (321) was proportionally allocated to the number of households identified. Finally; women from each kebele were included in the study by systematic sampling technique using a sampling interval of 10 units. The first sample to be included was identified using lottery method. When two or more eligible women were present in one household, only one woman was considered by lottery method.

#### Data collection

After reviewing different literatures; properly designed semi-structured questionnaire containing both close and open ended questions was developed in English to collect the data. The questionnaire contained a total of 38 questions and designed to collect data concerning sociodemographics (17 questions), fertility related factors (8 questions) and method related factors (13 questions). Data was collected for a period of 1 month by home to home visit using face to face interview. Four BSc health extension workers and two BSc midwives were involved in the data collection process as data collectors and supervisors respectively.

The quality of data maintained through the following measures: Translation of questionnaire to the local language, training of data collectors and supervisors on the purpose of the study and data collection process, close supervision and prompt feedback, revision of completed data every day, conducting pre-test before data collection.

#### Data management and analysis

Data was checked for completeness, the corresponding code number was written carefully at each margin. Data was entered and cleaned with EPI Info version 7 then exported to SPSS (V-23) for analysis. Modern contraceptive discontinuation has been defined as women who used condom, OCP, injectable, implants and IUCD contraception method in the past 5 years but were not using a method at the time of the survey [6].

Descriptive statistics such as mean (and median for skewed distribution) and quartiles were used to assess the general characteristics of participants and their current modern contraceptive status. Binary logistic regression was used to show crude association among dependent and independent variables. Finally, multiple logistic regression analysis was used to analyze the influences of socioeconomic, fertility and method related factors on modern contraceptive discontinuation by controlling confounding factors. Results were presented using text, figures and tables accordingly. *P*-value of < 0.05 was considered to determine statistical significance for both binary and multiple logistic regression.

## Results

### Socio-demographic and economic characteristics

This study was conducted among 321 married reproductive age women and the response rate was 100%. The mean age of participants was 29.6 years (SD + 5.6), where above one third of participants 119(37%), were in the age group between 25 and 29 years. Most of the participants 283(88.2%) were Orthodox by religion and Tigray 251(78.2%) by ethnicity. Nearly three fourth of the participants 228(71%) were housewives, and nearly half 135(42%) of their partners were farmers by occupation.

Majority of the participants 307(95.6%) were living with their husbands but the rest 14(4.4%) were not, and 19(5.9%) of participants reported that their husband had another wife, where one third 6 (31.6%) had two another wives. Majority of the participants 278(86.6%) were discussed with their female friends on MC and about 284(88.5%) stated the presence of husband support to use MC. Most of the participants 275(85.7%) reported they have television and nearly half 123(38.3%) have a radio in their home (Table 1).

**Table 1:** Socio-demographic and economic characteristics of participants, in Humera town, 2017 (n=321)

Variables	Category	N	Percent
Participants religion	Orthodox	283	88.2%
	Muslim	28	8.7%
	Protestant	7	2.2%
	Catholic	3	0.9%
Participants age (yr.)	<=24	53	16.5%
	25–29	119	37.1%
	30–34	80	24.9%
	> 35	69	21.5%
Educational status	Not able to read and write	25	7.8%
	Able to read and write	48	15.0%
	Primary school (1-8)	167	52.0%
	Secondary school (9-12)	66	20.6%
	College and above	15	4.7%
Occupation	Housewife	228	71.0%
	Government employee	24	7.5%
	Self-employee	69	21.5%
Husband educational level	Not able to read and write	23	7.2%
	Able to read and write	31	9.7%
	Primary school (1-8)	167	52.0%
	Secondary school (9-12)	67	20.9%
	College and above	33	10.3%
Husband occupation	Government employee	54	16.8%
	Self-employed	129	40.2%
	Farmer	135	42.1%
	Others^a^	3	0.9%
Mothers monthly income	No income	228	71%
	<=499 birr	18	5.6%
	500–999 birr	11	3.4%
	1000-1499birr	8	2.5%
	> = 1500 birr	56	17.5%
Husband monthly income	<=499birr	16	5.0%
	500–999 birr	11	3.4%
	1000-1499birr	21	6.5%
	> = 1500 birr	273	85.0%

^a^driver, barber

### Reproductive characteristics

Age at first marriage range was from 11 to 27 years with the mean age of 18.6 years (SD + 2.74). Majority of the participants 298 (92.8%) reported that they had an experience of giving birth and at first birth was 20 years (SD + 2.6) which ranges from 15 to 30 years. The average number of children per household was two (2.18) children (SD + 1.4).

Regarding desire to have additional children, of those who had experience of birth, 195(65.4%) reported that their husbands need to have additional children. Moreover, the number of desired children ranges from one child to seven children with an average of nearly four (3.87) children per household. Decision on the number of children mostly made by both husband and wife 286(89.1%), husband alone 24(7.48%), wife alone 10(3.12%) and other family members 1(0.31%).

### Prevalence of modern contraceptive discontinuation

The prevalence of modern contraceptive discontinuation was 27.1% (95% CI = 22.24, 31.96). Of those participants included in this study 234 (72.9%) were on modern contraceptive use during the interview time. Among the major MC methods, the highest discontinuation was for the OCP users (37.5%), and in contrast IUCD had the lowest discontinuation (14.3%) (Table 2).

**Table 2:** Variability of MC discontinuation by type of MC, among participants in Humera town, 2017(*n* = 321)

	Current status of MC use		Total
	Yes	No	
Type of MC used			
Condom	3 (75%)	1 (25%)	4
OCP	10 (62.5%)	6 (37.5%)	16
Injectable	134 (68.3%)	60 (30.9%)	194
Implant	81 (81%)	19 (19%)	100
IUCD	6 (85.7%)	1 (14.3%)	7
Total	234	87	321

The median duration of use before stopping the method was 27(IQR=27) months with minimum 1 and maximum 156 months. The discontinuation rate decreases with increase in duration of contraceptive use. The chances of discontinuation of modern contraceptive use are higher (40.7%) in the initial 1-6 months period and chances to dropout decrease to 27.8% during 7-12 months period (Table 3).

**Table 3:** Variability of MC discontinuation by the duration of MC use, among participants in Humera town, 2017(*n* = 321)

	Current status of MC use		Total
	Yes	No	
Duration of MC use			
<=6 month	16 (59.3%)	11 (40.7%)	27
7–12 month	39 (72.2%)	15 (27.8%)	54
> = 13 month	179 (74.6%)	61 (25.4%)	240
Total	234	87	321

Above half 58(66.7%) of the participants stated that want to get pregnant was their main reason for not currently using MC followed by experience of side effect 17(19.5%) (Table 4).

**Table 4:** Reasons for MC discontinuation, among participants in Humera town, 2017 (*n* = 87)

Reasons for MC discontinuation	Frequency(n)	Percent (%)
To get pregnant	58	66.7%
Side effect	17	19.5%
Partner disapproval	11	12.6%
Bad rumors	11	12.6%
Others^a^	9	10.3%

^a^fear of infertility, being menopause, to use other than MC, perceives MC is sinful

**Fig. 1:**
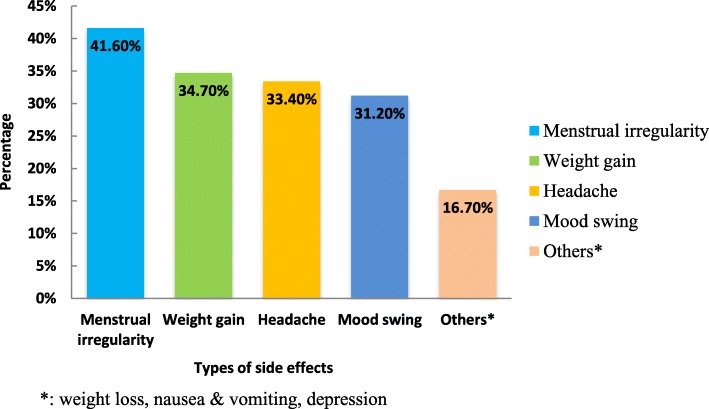
MC side effects experienced by participants in Humera town, 2017 (*n* = 234). Types of side effects (**%**) reported by women who experienced side effects of modern contraceptive method (234) analyzed using multiple response sets

Among those who experience side effects of MC (234), 73(31.1%) of participants discontinued using modern contraception. The most frequently reported side effect was menstrual irregularity (41.6%) followed by weight gain (34.7%) (Fig. 1).

All most all 319(99.4%) of participants had access to all type of modern contraceptive (MC). Of all participants, 319(99.4%) were utilizing the type of MC that they prefer to use and similarly most participants 292(91%) were counseled on side effects of contraceptive. Almost all 320(99.7%) participants reported that using MC has health benefits and 7(2.2%) of participants have stated that MC is harmful to the womb. Fifty five (17.1%) of the participants stated that there are things that prohibit them from using MC in their community.

### Factors associated with modern contraceptive discontinuation

On multiple logistic regressions analysis; experience of side effect (AOR = 3.01 CI = 1.2, 7.58), not discussing on MC with female friend (AOR = 3.26 CI = 1.27, 8.36), not counseled on side effects (AOR = 6.55 CI = 2.21, 19.39), haven’t had husband support to use MC (AOR = 12.99 CI = 4.59, 36.78) community prohibition to use MC (AOR = 6.88 CI = 3.05, 15.51), having less than two male children (AOR = 2.51 CI = 1.06, 5.96) and desired number of children (AOR = 2.83 CI = 1.16, 6.89) were found to be predictors at < 0.05 *p*-value significance level (Table 5).

## Discussion

This study was conducted to assess discontinuation of modern contraception among married women of reproductive age group who live in Humera town, North Ethiopia. In this study, the prevalence of MC discontinuation among married women who had a history of MC use within 5 years at the time of survey was 27.1% with a median duration of 27 months. This is consistent with other studies conducted in Peshawar (27.5%) and Agarfa (25.5%) districts [3, 8]. This similarity might be due to the fact that these studies conducted in a specific community and the educational level of most study participants were primary schools and above. On the other hand, this finding is lower than study findings in Bangladesh (36%), Philippines (37.2%), Colombia (43%), Kenya (33–36%) and Ethiopia (37.1%) [6, 9–11]. This could be because of different reasons; one and main reason might be due to the study period, as the current contraceptive use service has improved compared to other studies. The other possible reason might be the study population; the studies done in the countries mentioned above were done at national level with high sample size and included both rural and urban participants.

**Table 5:** Multivariate analyses of selected factors affecting MC discontinuation among married women in the reproductive age group, Humera town, 2017 (*n* = 321)

Variables	Current status of MC use		COR (95%CI)	AOR (95%CI)	*P*- value
	Yes	No			
Husband wants to have an additional child					
Yes	138	57	2.62 (1.38,4.99)	1.26 (.50,3.19)	0.62
No	89	14	1	1	
Using implant type of MC					
Yes	82	19	.52 (029,.92)	0.77 (.36,1.67)	0.51
No	152	68	1	1	
Number of desired children					
< =3	91	19	1	1	
> =4	143	68	2.28 (1.28,4.04)	2.83 (1.16,6.89)	0.02*
Experience of side effects					
Yes	161	73	2.36 (1.25,4.46)	3.01 (1.2,7.58)	0.01*
No	73	14	1	1	
Discussion on MC with a female friend					
Yes	214	64	1	1	
No	20	23	3.84 (1.98,7.44)	3.26 (1.27,8.36)	0.01*
Counseled on side effects					
Yes	226	66	1	1	
No	8	21	8.98 (3.81,21.22)	6.55 (2.21,19.39)	0.00*
Number of alive male children					
0–1 male	148	57	2.17 (1.14,4.14)	2.51 (1.06,5.96)	0.03*
> = 2 males	79	14	1	1	
Husband support to use MC					
Yes	226	58	1	1	
No	8	29	14.12 (6.13,32.53)	12.99 (4.59,36.78)	0.00*
Presence of community prohibition					
Yes	20	35	7.20 (3.84,13.48)	6.88 (3.05,15.51)	0.00*
No	214	52	1	1	

*Statistical significant at *P*-value < 0.05

In this study we asked participants reason for MCD. Above half (66.7%) of discontinuers stated that desire to get pregnant was their main reason for not using MC followed by experience of side effect (19.5%) and partner disapproval (12.6%). Similar reasons for discontinuation were reported by studies done in Agarfa, Ethiopia and Bangladesh [3, 11]. Contrary to this other studies reported different reasons: in Ofla district, Tigray, Ethiopia; health concern (46.2%), want more children (43.6%) and side effect (35.9%), in Philippines method failure (14%), side effects (6.2%) and to get pregnant (5.6%) and in Peshawar health concern (45.5%), side effect (40%) and wanted more children (34.5%) [8, 9, 12]. This difference with a study in Ofla district might be due to different population characteristics since it was done in a district as compared to this study which is done in a town. On the other hand, the difference with a study done in Philippines and Peshawar could be because they are developed country and may be more aware of MC use.

Our findings concerning factors associated with modern contraceptive discontinuation generally corroborate with previous research. This study shows that desire to have four or more children increase odd of modern contraceptive discontinuation [13]. In line to this; a study done in Armenia reported that odds of discontinuation increase nearly two times if a women’s number of live children were four or more as compared to women who had less than four children [14] and in Agarfa district for a single increase in family size the likelihood of MC discontinuation decreases by 12 % [3].

This study identified that not having male child or having only one alive male child increase the chance of modern contraceptive discontinuation, which is a new finding identified only in this study. This might be due to wrong community perception still there is a gap in believing gender equality, that is why even though they have more children but they want to have a male child.

This study also found that experiencing contraceptive side effects as one of the major reason for contraceptive discontinuation and experience of contraceptive side effect of particular contraceptive method was found to be strongly associated with MCD that increase the chance of discontinuation by three fold. Consistent to this finding a study in Peshawar reported that women who experience side effects were eight times more likely to discontinue using contraception [8] and in Ofla, Tigray, Ethiopia; where women who have experienced side effects after insertion of Implanon were 2.8 times more likely to discontinue as compared to women who didn’t [12]. One and main reason might be due to poor pre dispensation counseling on the commonly happened side effects and strategies for method mix or switching in case of experiencing a side effect.

Improving quality of contraceptive counseling is one strategy to decrease contraceptive discontinuation and hence reduce unintended pregnancy. Women should routinely be informed about side effects during counseling and offered a possibility to switch methods if necessary during counseling. This study identified that women who didn’t get pre dispensation counseling on side effects of MC were at high chance to discontinue a particular contraceptive method. Contrary to this pre dispensation counseling on side effects was not reported as predictor of modern contraceptive discontinuation [5]. This might be related to the fact that inadequate pre dispensation counseling about the main and possible side effects of MC by service providers lead to early discontinuation, as users may not be familiar what will happen related to the method and they may not ready for the solutions as well.

Findings of this study also showed that not discussing on MC with female friend increase odds of discontinuation by three fold. Consistent with this finding was reported by a study done in Agarfa district women who discussed on MC with their female friend decreases discontinuation by 90% as compared to were not discussed [3]. This might be because of high acceptance and trust of women for their female friends especially if they were satisfied users. The other possible reason could be in both studies, above one-third of the participants were in the age group of 25–29 years which is highly influenced by peer pressure.

Absence of husband support was found to be significantly associated with modern contraceptive discontinuation. This finding is in line with the study in Bangladesh where women who did not have their husbands’ support in their OCP use had double the risk of discontinuing OC use than their counterparts [11] and a study in Agarfa district women who had support from their husbands to use MC decreases MCD by 85% than those who had no support from their husbands [3]. The possible reason might be the fact that husband disapproval in using contraceptive is a reason for MCD. Moreover, in these studies most of the participant’s occupations were housewife and they could be economically dependent on husbands’ income which in turn influences their decision.

We also found that presence of community prohibition as another predictor of contraceptive discontinuation. The proportion of contraceptive discontinuation was significantly higher among women who reported presence of negative hearsay in their community regarding contraceptives. This finding agrees with study done in Agarfa district which shows high chance of contraceptive discontinuation among women reported presence of community prohibition [3]. The possible reason for this could be wrong perception and bad rumors of community on MC use, they perceive that it is sinful, cause’s infertility, interfere with sexual satisfaction and many others which will lead to increment in discontinuation rate.

### Strengths and limitations of the study

The strength of this study was being community-based study to be more representative and using interview for data collection method which has good reliability and excludes incomplete data. However, despite its strength, the study was not without limitations, since this study was limited to married women only at the time of the study; results may not be generalized to all women in Humera town.

## Conclusion

This study shows the magnitude of modern contraception discontinuation is high among reproductive age group women in Humera town. The number of desired children, number of alive male children, experience of side effects, discussion on MC with a female friend, counseled on side effects, husband support to use MC and presence of community prohibition from using MC were the predictors of modern contraception discontinuation. The health care providers should focus on pre provision counseling, partner involvement on decision making process. In addition to this great effort should be made to address community perceptions and understanding of benefit of modern contraceptives use.

## Abbreviations

AOR: Adjusted odds ratio
DHS: Demographic and health surveys
IUCD: Intrauterine contraceptive device
MC: Modern contraceptive
MCD: Modern contraceptive discontinuation
OCP: Oral contraceptive pills
SPSS: Statistical package for social science
